# Successes and Challenges From a Motivational Interviewing-Informed Diabetes Prevention Program Situated in the Community

**DOI:** 10.1177/15248399221115066

**Published:** 2022-09-01

**Authors:** Tineke E. Dineen, Corliss Bean, Mary E. Jung

**Affiliations:** 1 The University of British Columbia, Kelowna, British Columbia, Canada; 2 Brock University, St. Catharines, Ontario, Canada

**Keywords:** process evaluation, client receptivity, user-feedback, motivational interviewing, prediabetic state, qualitative methods

## Abstract

To manage the rising prevalence of type 2 diabetes mellitus, sustainable diabetes prevention programs are needed. In this study, a process evaluation was conducted to qualitatively understand perceived successes and challenges of a diabetes prevention program situated in the community. This study took place in the first year of a multiyear project. Semistructured interviews were conducted with a sample of women clients (n = 14) postprogram and trainers (n = 10) 9 months into program implementation. Interviews were audio-recoded, transcribed verbatim, and analyzed using a Template Approach. Data were first analyzed deductively into two categories that aligned with the study’s purpose (successes and challenges). Second, an inductive analysis was used to understand program delivery processes within each category. Clients and trainers expressed (a) program successes related to recruitment strategy, outlook on making behavior changes, and communication style used within the program and (b) program challenges surrounding effort of learning and applying the communication strategy, usefulness of program applications and tools, and program fit. This evaluation provides practical implications and future directions for diabetes prevention programs, and has informed tailoring and expansion of the program of focus. Results demonstrate the success of motivational interviewing from both client and trainer perspectives and the impact of community partnerships to increase prediabetes awareness in the community. Overall, the program’s diabetes prevention and behavior change strategies coupled with a client-centered approach facilitated women clients in making diet and exercise modifications.

Worldwide, the rate of individuals living with type 1 and type 2 diabetes mellitus (T2DM) is rising, having tripled in the past 20 years, with T2DM accounting for almost 90% of the cases (International Diabetes Federation, 2019). Multiple landmark clinical trials outlined the efficacy of diabetes prevention programs (DPPs) in reducing future T2DM risk through diet and exercise modifications (see Gruss et al., 2019). However, once completed, clients have struggled to adhere to diet and exercise changes in their daily lives (Knowler et al., 2009). Adherence is even lower for women. In a follow-up to the landmark United States DPP, women had significantly lower physical activity compared to men (Rockette-Wagner et al., 2017). Understanding ways to promote maintenance of diet and exercise adherence, particularly in women with prediabetes, are needed. Process evaluations can uncover the activities and conditions to understand *how* and *why* programs were effective or ineffective.

Small Steps for Big Changes (SSBC) is a brief motivational interviewing (MI)-informed DPP designed to empower clients to make diet and exercise changes that suit their lives. The program consists of a baseline appointment and six program sessions, each of which includes MI-informed behavioral (diet and exercise) counseling and supervised exercise (see Table 1 for program overview). The program encourages client autonomy and provides choice between exercise regimens (high-intensity interval training [HIIT] or moderate-intensity continuous training [MICT]). Home days encourage clients to independently work toward goals between sessions. This study was conducted at a community site, led by trainers from the research team (undergraduate students, graduate students, and postdoctoral fellows). To date, the overall demographic of those who expressed interest and enrolled in the program are predominantly women (70%; Bean et al., 2021). This is consistent with other community-based diet and exercise programs (e.g., 65% women; Zigmont et al., 2018).

**Table 1:** Program Overview by Session Number and Topic of Focus

*Week*	*Session number*	*Topic*
Week 1	1	Discovering why making diet and exercise changes is important to the client
	2	Exploring added sugar
	3	Understanding physical sensations during exercise
Week 2	4	Exploring carbohydrate choices
	5	Finding ways to exercise that work for the client’s lifestyle
Week 3	6	Managing slips throughout the client’s daily life

A key program component is MI, a communication style shown to be effective in helping individuals with T2DM make diet and exercise modifications (Christie & Channon, 2014). MI works through collaborative conversations to evoke a client’s motivation for change, thereby strengthening their commitment to change (Miller & Rollnick, 2013). Briefly, MI conversations center on four core relational elements (compassion, acceptance, partnership, and evocation) that make-up the overall spirit of MI (Miller & Rollnick, 2013). During a session, counselors use four communication skills (open-ended questions, affirmations, reflections, and summaries “OARS”) and work through four processes (engaging, focusing, evoking, and planning) to explore changes that clients would like to make. Used together, a skilled counselor will elicit *change talk*, a client’s argument for making change, and weaken *sustain talk*, a client’s arguments against making change (Miller & Rollnick, 2013). To date, limited research has examined receptivity of MI and no research has specifically examined women’s experiences with MI. One study qualitatively explored clients’ experiences after participating in four sessions of MI; however, the study only examined whether clients were aware of their therapist’s use of MI (Marcus et al., 2011). Another multimethod study included qualitative data from both clients and peer supporters; however, the study lacked an in-depth exploration of the acceptability of MI from clients’ perspectives (Copeland et al., 2018). Although MI has potential to positively impact clients’ behaviors, no studies to date have qualitatively explored *why* MI was effective. Furthermore, no study has examined the acceptability of MI from client and trainer perspectives in a DPP.

As such, the purpose of this study was to conduct a process evaluation to explore successes and challenges of an MI-informed program from client and trainer perspectives. Given the large number of women clients, and the limited qualitative work conducted in this area, the authors utilized a homogeneous sample of the program’s majority. Despite women’s predominance in DPPs, limited research has examined women-specific experiences of participating in such a program. Knowing there are differences in support strategies and the promotion of diet and exercise behaviors between men and women (Harreiter & Kautzky-Willer, 2018; Kawachi & Berkman, 2001), an in-depth exploration into women’s experiences in a DPP is warranted.

## Methodology

### Paradigmatic Position

This research was guided by a pragmatic epistemology, which seeks to develop practical recommendations and tolerates multiple truths or realities (Dewey, 1931). Although all trainers were exposed to the same training, and all women clients were exposed to the same program, everyone has unique experiences. A qualitative description methodological approach (Sandelowski, 2010) was used to understand women clients’ and trainers’ experiences in the program. This approach is useful to understand the perspective of those involved in a phenomenon and is well-suited for a pragmatic epistemology.

### Participants and Procedure

This study was part of a larger, ongoing project that investigated the effectiveness of SSBC in a community setting. This qualitative study was conducted within the first year of the 3-year project, an ideal time to gain insight into the program processes, obtain feedback, and make necessary changes to improve program implementation. Prior to data collection, ethical approval was obtained from the researchers’ institution. This study involved two sets of participants: (a) women clients who engaged in SSBC and (b) trainers who delivered SSBC. Clients were recruited through various recruitment strategies (e.g., local medical laboratory referral system, online forums, promotional materials, and events; see Bean et al., [2021] for full description). All women entering the program between May 2017 and January 2018 were asked at program intake to engage in an interview postprogram. Fourteen women agreed and interviews took place within 5 days postprogram. Clients ranged from 48 to 65 years old (*M* = 60.13, *SD* = 4.87) and all identified as Caucasian. All trainers (*n* = 10) who were trained during the aforementioned period and counseled at least one client through the program were invited, and agreed, to participate in an interview between 6 and 11 months into program implementation (*M* = 9 months). Such timing ensured trainers had experienced facilitating clients through the program, and interviews were held within the first year of the study. The 10 trainers ranged from 22 to 30 years old (*M* = 25.1, *SD* = 2.6), 70% identified as women, and 70% identified as White. A detailed description of the training protocol is described elsewhere (Cranston et al., submitted). Briefly, the training covered SSBC program content and core components of MI taught using a variety of didactic and experiential learning. The training covered the spirit of MI, the four processes, and four communication skills. An expert trainer shadowed all trainees throughout their first client and provided feedback. A detailed fidelity assessment of MI using the Motivational Interviewing Competency Assessment (MICA) demonstrated that on average trainers were operating at a client-centered level of MI and retained those levels 3 months posttraining (Cranston et al., submitted). All interviews were conducted by the first or second authors after written informed consent was obtained. Interview options (in-person [*n* = 8], telephone [*n* = 16]), were provided to prioritize participant convenience.

Separate interview guides were developed for clients and trainers based on program content/resources, training content/resources, and an extensive literature review (e.g., Matthews et al., 2014; Tulloch et al., 2013). Interview questions explored clients’ experiences engaging in the program and trainers’ experiences delivering the program (see Table 2 for sample questions). All interviews were audio-recorded, transcribed verbatim, and reviewed by the first author. Client interviews lasted 51 to 76 minutes (*M* = 54 minutes) and trainer interviews lasted 31 to 82 minutes (*M* = 58 minutes). To increase data trustworthiness, transcripts were sent to clients for member checking (Creswell & Miller, 2000), and results were triangulated from two perspectives. Clients received a $20 honorarium for interview participation.

**Table 2:** Sample Interview Guide Questions

*Interview participant*	*Interview questions*
Client	How did you find SSBC? What did you like about the program? Dislike? What has your experience been like working with the trainers? What have you learned from being involved in the program? What do you like most about the exercise/nutrition component of the program? What challenges have you experienced? What recommendations for you have for improving the program?
Trainer	Tell me about your experience as a trainer for SSBC? How satisfied were you with the overall training experience? Did you feel well-prepared for when you had your first independent session? Why/why not? What could make you feel more prepared? What do you feel is working well for you when you are training clients? What challenges have you experienced? What recommendations do you have for training? Program delivery?

### Data Analysis

A Template Approach (King, 2004) was used to thematically analyze interview transcripts. NVivo12 software (QRS International Pty Ltd, 2018) facilitated data coding and organization. To ensure consistent and reliable coding, after familiarization with the data, the first and second authors independently coded two client and two trainer transcripts and met to discuss themes and subthemes. Together, these two authors developed an initial coding template guided by the study’s purpose. The coding template included two deductively coded categories (successes and challenges), with inductive codes generated in each category. The first author applied the developed template to the remaining transcripts. Iterative changes were made to the template to comprehensively represent the data as new transcripts were analyzed. The second and third authors iteratively reviewed and discussed the template with the first author, which led to multiple rounds of analyses. Coding discrepancies were discussed between authors until consensus was reached. Main themes were identified, and relevant quotations were selected to accurately represent each theme. In line with a qualitative descriptive methodology, the analysis described experiences and stayed close to the words used from the participants’ experiences.

## Results

### Program Successes

Clients and trainers all described generally positive program experiences. Four themes were generated in relation to program successes: (a) program recruitment fostered prediabetes awareness, (b) program engagement facilitated a new outlook on behavior change, (c) integration of MI techniques were well-received by clients, and (d) learning and applying diabetes prevention strategies in the real-world. Each theme is discussed in detail below and participants’ quotes are used to support each theme (Table 3).

**Table 3:** Overview of Categories, Themes, and Example Quotes From Both Trainers and Clients Participating in a Diabetes Prevention Program Situated in the Community

*Category*	*Theme*	*Example quote*
Program successes	Program recruitment fostered prediabetes awareness	“I got a letter from the (medical) lab[oratory]. . . At first, I went, really? Because I think of myself as fairly healthy to start with. . .then when I started looking into it, I understood more of what it was. It’s not diabetes per say, but definitely a warning sign. Then, when I started keeping track of everything, I went ‘oh yeah maybe I’m eating too much sugar and too much other stuff so’. . . awareness.” (C5)
		“I’ve mentioned to people that prediabetes is actually a diagnosis and they go, ‘You’re kidding!’; they had no idea.” (C15)
	Program engagement facilitated a new outlook on behavior change	“I can’t just do it to feel good now; I have to do it for my health. That’s what the program gets through to you.” (C6)
		“I have hope that I am not going to become diabetic. Where I felt before that that was inevitable” (C1)
		“If you think that you’re supposed to do something, and you don’t do it then you feel really bad and then you go: ‘to heck with it’. Just [trainer] going through how you change your thought patterns to be more positive, forgiving yourself, letting it go, starting a new day.” (C5)
		“How much people worry about the stigma surrounding type 2 [diabetes]. . .which is upsetting. It’s not just a physical disease, but a lot of them struggle more with the mental aspect of it. . . That’s the biggest thing I’ve learned: just how important the counselling session was.” (T5)
	Integration of MI techniques were well-received by clients	“[Trainer]’s questions were always open-ended, like ‘how do you think you could do this?’ or ‘what do you think could be different?’. . . it pushes you to think about goals and about options.” (C8)
		“When you’re telling it to yourself you can easily dismiss, right? But if somebody else is saying ‘no, look what you’ve achieved, you go. . .hey yeah’.” (C1)
		“Nothing was ever negative and they never used criticism.” (C19)
		“[I liked] how they didn’t think anything was stupid.” (C6)
		“I felt very supported. I felt that I had the opportunity to explore some things that I had never really. . .or hadn’t vocalized for a while.” (C8)
		“I’ve had a few participants say like, ‘Oh I love the way we do these sessions and I just get to talk, and I get to tell you about things, and we get to work through making goals.’ And I think that’s all due to MI [motivational interviewing].” (T6)
		“I think it’s a huge impact on their outcomes because it’s ultimately them and their ideas and it’s not really in our position to be like, ‘You have to do this to change this lifestyle modification for you’, so I think it’s actually a really big impact on their outcome because it’s what fits in their lifestyle and their ideas. What will suit them best.” (T9)
		“. . .[MI] is absolutely amazing because when you do use them [OARS], clients just open up.” (T7)
		“Rather than thinking about what I want to cover next, really listening to them because I find that eventually you do cover all the points you need to cover and allowing conversation to take shape that way.” (T8)
	Learning and applying diabetes prevention strategies in the real world	“One thing I’ve never ever looked for is added sugars in things. It floored me what products we have around the house that would use up your whole daily intake of added sugars in one serving.” (C12)
		“I’ve heavily reduced my carbohydrates, [yet] I’m still having carbohydrates because I know they’re important, but I’m having the good kind—rolled oats, oat bran, vegetables. . .rather than having two cookies for a snack, I’m having a small apple.” (C17)
		“We have a hill nearby, so I just walk the hill. . .Another day I’ll go to our gym on the stationary bike and [do] intervals. The days that I don’t want to go outside, I’ve got a Wii and do Zumba. . .different things that I can do all the time that will keep me motivated and interested to do it.” (C12)
		“I was doing intensive interval training, which I like[d] because it was quick and dirty, and I’m done.” (C1)
		“I really liked tracking because I could keep my sugars under control” (C20)
		“I really enjoyed learning more about where my heart rate should be when I’m working out. In the past, I would think ‘I’m working too hard, my heart is getting too stressed, I’d better slow down’, but in fact, it’s good to get it to that level.” (C17)
Program challenges	Ongoing effort of learning and applying MI	“There was a lot going on and to remember in the back of your head. . .a lot of the time, I would just be too much in the back of my head so then I would just blurt something out.” (T3)
		“Initially, I think I over-thought the process too much, and now, I think maybe it comes a bit more naturally. . . I’m starting to think maybe I should go back to overthinking to be on my toes.” (T9)
		“When a person brings something up—a behavior that’s not part of the program—that they want to change that we can be reasonably certain, if they want to continue, it isn’t going to lead to diabetes prevention.” (T1)
	Usefulness of program applications and tools	“I don’t like the heart rate monitor because I have that on the bike anyway. I found that it just made me nuts with all its beeping and squeaking.” (C8)
		“I find a lot of the products very American. Finding comparable products is a little hard.” (C10)
		“I would prefer if it was in ounces because it’s easier to have a scale, or even on the package it’s usually grams.” (C14)
		“I started using it [program workbook] a little bit and then I kind of didn’t.” (C19)
		“The notebook [program workbook] that we provide them, I don’t know if any of my clients actually used those. They don’t tell me that they’re not using them, I just get a sense that they’re not using them.” (T11)
	Program fit	“I started off at 20 [minutes], and that’s why I think this is built for people who are the entry level of physical activity because when I go walking with the group, we’re out for an hour and a half”.” (C20)
		“Sometimes clients join the program and I don’t think they always know why they joined the program. I’ve had some [clients] who are fine with exercising, fine with eating and that’s challenging.” (T8)

*Note*. MI = motivational interviewing; OARS = open-ended questions, affirmations, reflections, summaries.

#### Program Recruitment Fostered Prediabetes Awareness

Several clients discovered they were at risk for developing T2DM through the study’s primary recruitment method (receiving a study information letter from a local medical laboratory after client’s bloodwork fell within the diabetes risk range [A1C of 5.7%–6.4%]). Clients discussed how receiving the letter prompted them to reflect on their health, become motivated to change, and empowered them to learn more about prediabetes and their A1C results. By learning more about prediabetes and their risk of developing T2DM, clients shared this information with others with the goal of raising prediabetes awareness.

#### Program Engagement Facilitated a New Outlook on Behavior Change

Upon completing the program, many clients described a new focus on making diet and exercise modifications to benefit their health. This new health-focused outlook contrasts with their previous attempts at making diet and exercise modifications, which focused more on physical appearance. Clients also felt more in control over whether they would develop T2DM. This sense of control motivated clients to prioritize diet and exercise modifications with the goal of reducing their risk of T2DM.

Almost all women discussed adopting a new self-compassionate outlook on themselves and their efforts to make diet and exercise modifications. The women mentioned not wanting to beat themselves up for a slip but rather focus on what they could do the following day to prioritize their health. Clients viewed this forgiving outlook as something they learned from program participation. The value of adopting a self-compassionate outlook became clear for both clients and trainers. From one trainer’s perspective, it was eye-opening to witness the mental stress some clients experienced and reinforced the importance of counseling sessions that helped clients focus on strengths and hard work. Clients linked these two new outlooks with sustaining a positive attitude for behavior change maintenance.

#### Integration of MI Techniques Were Well-Received by Clients

The use and impact of the four MI micro-skills (open-ended questions, affirmations, reflections, and summaries; Miller & Rollnick, 2013) facilitated a client-centered approach. When using OARS, trainers felt they were able to engage clients in introspective discussions and make them feel comfortable. This approach helped clients open up, reflect, and create their own solutions to situations. Trainers discussed the impact of a well-placed affirmation, as opposed to praise, to provide effective confidence boosts that supported and ensured clients recognized their own efforts. The impact of affirmations was also evident from the clients’ perspectives, as one client described how the trainer helped her see her accomplishments more clearly.

Trainers were taught to use MI skills to evoke ideas that came from the client, provide options, and prioritize client choice. This approach allowed clients to guide the session and collaborate with their trainers to find meaningful solutions for their personal situations. From the trainers’ perspectives, the use of MI skills offered clients ownership over their goals and provided space for clients to figure out what would realistically work for them. This approach helped strengthen a client’s commitment to change. Clients felt that trainers genuinely wanted to understand them, and their situation, and valued the empathic, nonjudgmental nature of their trainers. The open, supportive nature of conversations enabled clients to gain confidence and trust in sharing details with their trainer they might not have been comfortable saying. The one-on-one nature of the program was perceived to help foster this supportive environment.

#### Learning and Applying Diabetes Prevention Strategies in the Real World

Clients learned about relevant diabetes risk reducing strategies through diet and exercise behavior change. The information provided new awareness around sugars, carbohydrates, and exercise approaches. Clients often discussed dietary changes they had already made and/or planned to make. The health tracking and heart rate applications supported the program; encouraged clients to self-monitor their behaviors; and helped clients set, track, and stay accountable to their goals. Clients enjoyed learning about exercising at high intensities with HIIT, which was identified as a new exercise approach for many, and enjoyed being given a personalized target heart rate zone to tailor their exercise sessions. Many clients spoke that having a target heart rate facilitated self-monitoring of their exercise intensity and provided a goal to achieve during their exercise bouts. In between sessions, trainers encouraged clients to discover exercise opportunities that worked for them on their own time (e.g., fitness facility, at home, or in their neighborhoods). These home days helped clients find exercise options in their everyday environments to enact what they learned in the real world.

### Program Challenges

Challenges related to program delivery were identified, including: (a) ongoing effort of learning and applying MI, (b) usefulness of program applications and tools, and (c) program fit.

#### Ongoing Effort of Learning and Applying MI

MI was designed as a central component of the SSBC program and emerged, through the qualitative analysis, as critical to the experiences of both clients and trainers. Specifically, trainers felt they connected and progressed their client the most during sessions when they effectively used MI skills. However, trainers found learning MI to be challenging. MI was not perceived as an intuitive communication style; it required cognitive effort to use MI during a session. As trainers gained more experience, they reported thinking less about MI during their sessions. However, there was a potential trade-off between having more natural sessions and being MI-conscientious. At times, trainers found it difficult to balance SSBC content with the client-centered approach. Using a client-centered approach, trainers wanted to support clients’ autonomy; however, situations emerged when clients’ choices were not in line with diabetes risk reduction. Trainers recognized the importance of MI to program success and suggested having ongoing MI skills training, ongoing feedback on their MI skills in practice, or booster sessions as ways to maintain MI skills over time.

#### Usefulness of Program Applications and Tools

Although the program applications (heart rate and health tracking) were primarily seen as valuable for clients, there were also perceived challenges associated with them. For some clients, the applications were not seen as helpful as they had other means to access similar information (e.g., using heart rate sensors on fitness equipment, their personal heart rate watch, or a different diet tracking application). Specifically concerning the diet tracking application, complaints centered around the time-consuming nature of entering food and the challenge in locating Canadian products or calculating metric serving sizes, as the application was American. Finally, some clients did not find the program workbook useful; many used it to document a few notes but did not use it beyond that. These observations were reinforced by the trainers’ reflections. Ideas to increase usefulness of the workbook included providing additional diabetes-related information, increasing interactivity of the workbook, and allowing choice in the use of heart rate watch/diet tracking application.

#### Program Fit

Some clients felt the program was not a perfect fit. Although incoming clients had an A1C that indicated they were at risk of developing T2DM, some clients perceived they were already practicing healthy diet and exercise behaviors. A few clients mentioned that the exercise component was too easy for them. Trainers found it challenging to work with clients who were already physically active and/or had a nutritious diet. Despite the program not always being a perfect fit, those interviewed still discussed benefits of enrolling in the program, such as gaining confidence in their health behaviors and increasing their awareness through tracking their diet and exercise.

## Discussion

The purpose of this process evaluation was to qualitatively explore successes and challenges of an MI-informed program from client and trainer experiences. Building on prior research demonstrating preliminary effectiveness of Small Steps for Big Changes in a community setting (Bean et al., 2021), this study supports program acceptability and triangulated results from two perspectives. Participants identified successes related to the program’s recruitment strategy, content, and use of MI. Program challenges were identified related to usefulness of program applications and tools, MI, and program fit. The evaluation results have practical and research implications leading to recommendations for both Small Steps for Big Changes and other organizations looking to facilitate a DPP.

Receiving the program information letter from the local medical laboratory referral system was the first time many clients learned of prediabetes and their risk for developing T2DM. Clients spread this new knowledge to their social networks (e.g., family, friends, and physician). As such, the main program recruitment strategy resulted in a valuable health promotion strategy. Early detection and management of prediabetes is imperative (Koopman et al., 2006). Prediabetes has an asymptomatic preclinical phase, making it hard for individuals to be aware of their condition, and health complications can already begin developing (M. I. Harris & Eastman, 2000; Koopman et al., 2006). Prediabetes awareness is a crucial step in helping reduce the burden of T2DM. Within a recent study, only 15.3% of American adults with prediabetes reported being told by their health care professional that they were at risk for developing T2DM (Centers for Disease Control and Prevention, 2020). Laboratory referral systems that promote prediabetes awareness are worthwhile program recruitment strategies to explore.

Small Steps for Big Changes fostered new outlooks on behavior change. Reframing to focus on health has been previously documented in weight loss counseling, where dieting or weight loss was associated with negative emotions, deprivation, and short-term efforts; focusing on health-supported long-term efforts (Hartmann-Boyce et al., 2018). This focus on health may be especially useful for women who are overweight with a history of chronic dieting (Penney & Kirk, 2015). Negative emotions from failed efforts can compromise self-regulation (Gilbert et al., 2010), a necessary skill to behavior change that works through activities such as goal setting and coping with setbacks (Terry & Leary, 2011). Adopting a self-compassionate outlook may offer helpful ways to deal with negative emotions, health threats, and failed efforts (Friis et al., 2015). This outlook is meaningful as women tend to have lower levels of self-compassion compared to men (Neff & Vonk, 2009). Many women clients learned to adopt a self-compassionate outlook. If something did not go as planned, they practiced letting it go and getting back on track. Diet and exercise behavior change is a long-term commitment with inevitable setbacks; adopting a self-compassionate outlook may help cope with these setbacks and promote maintenance of behavior change (Terry & Leary, 2011).

MI emerged as a potential mechanism of action to facilitate new outlooks on self-compassion and health. MI has been identified as a promising approach to enhance self-compassion (Steindl et al., 2018). Similarly, the appropriate use of MI to support clients’ personal reasons for change may have facilitated clients’ value on health as opposed to physical appearance (Miller & Rollnick, 2013). The use of the spirit of MI (compassion, accepting, partnering, and empathy) and communication skills (OARS) were evident from both trainers and clients. While this research did not explore the underlying mechanisms of MI, findings suggest both technical (demonstration of MI skills) and relational (use of the spirit of MI) skills are important. This finding is in line with Miller and Rollnick’s (2013) conceptualization whereby creating a supportive, safe environment through the spirit of MI may have enabled individuals to open up more in response to OARS. Technical skills have demonstrated support as a mechanism of MI with relational skills having fewer clear results (Magill et al., 2018; Pace et al., 2017). Recent research has also demonstrated the importance of both skills in MI communication (Villarosa-Hurlocker et al., 2019). Comparable findings have been shown in interventions using MI in primary health care and with individuals living with anxiety disorders, whereby clients described trainers as being empathic, providing a safe place to speak, using a nonconfrontational approach, providing affirmation, and increasing motivation for change (Brobeck et al., 2011; Marcus et al., 2011). Future DPPs should consider adopting an individual, MI-approach.

Clients valued the information and behavior change strategies provided through the program. Although program applications created some challenges (i.e., issues with the technology, time-consuming, and measurement units), they also complemented the program, with clients opting to continue using the applications postprogram. Program applications enabled clients to self-monitor behaviors, gauge goal achievement, and modify goals as needed. Self-monitoring and action planning are common constructs reported to significantly influence the effectiveness of physical activity interventions (Michie et al., 2009). Program applications were chosen for research purposes as user analytic data could be securely transferred to the research group. The researchers did not anticipate significant challenges with using an American tracking application since many American products are found in the Canadian market, the application included options to track generic products or manually enter products, and the majority of Canadians are familiar with quantifying foods in American metrics. In practice, this rigidity in predetermined applications could have been a barrier for some clients and demonstrates the tension between research and real-world community programs. From an MI-perspective, it is futile to ask an individual to complete a task they are not motivated to do (Miller & Rollnick, 2013). To support a client’s autonomy, trainers are encouraged to teach relevant self-regulatory skills without the program application or use a client’s preferred application. Finally, the program workbook was modified to be more interactive with program content and now includes a self-monitoring section and diabetes-related information.

### Limitations

Although this was one of the first studies to qualitatively assess the delivery of an MI-informed DPP from two perspectives (clients and trainers), study limitations exist. The first author acted as both program coordinator and researcher, which commonly occurs within community-based programs (M. I. Harris & Eastman, 2000). Internal evaluators may be less objective and introduce positive bias (M. J. Harris, 2016). However, having an internal evaluator allows for in-depth exploration of program components through data collection and analysis, which inevitably leads to valuable insights as well as biases (M. I. Harris & Eastman, 2000). Second, this study represented only women clients’ perspectives who completed the program and at one timepoint. Despite the high number of women clients (70%) and program-completion rate (95%; Bean et al., 2021), future research is needed to explore the experiences from other identified genders and those who withdrew from the program.

### Implications

This research demonstrates Small Steps for Big Changes is acceptable from clients’ and trainers’ perspectives, facilitated behavior change, and directly informed program modifications (e.g., program workbook). Conducting a qualitative process evaluation within a new program provides in-depth insight into program implementation and receipt that can be used to improve and adapt the program. Results uncovered unintended effects such as the impact of an American diet tracking application. As programs move from tightly controlled research laboratories to the community, program developers must find a balance between making decisions for research and community practicality. Findings can inform other research and practice efforts in diabetes prevention. Specifically, results encourage community partnerships to increase prediabetes awareness through recruitment referral systems and community organizations to support scale-up. In addition, MI emerged as a factor that facilitated counseling sessions and may support the maintenance of behavior change. Future research should explore the use of MI to facilitate long-term behavior change, and future programs should consider integrating this client-centered communication style. To impact more individuals at risk of developing T2DM, future work will scale-up Small Steps for Big Changes. Capitalizing on a research–community partnership, the community partner, who is a leader in community programming, can provide expansion venues, enhanced reach, sustainability, and support. As new sites develop our community partner provides local knowledge, community insight, and informal feedback to increase the likelihood of program success within each new context. As the prevalence of T2DM continues to rise, prediabetes awareness, along with sustainable and effective DPPs, are needed to empower individuals to reduce their risk.
